# A study of high-speed train delays and relevant propagation influence characteristics

**DOI:** 10.1371/journal.pone.0314293

**Published:** 2025-02-11

**Authors:** Jingyi Qin, Jun Zhang, Shuyao Wu, Shejun Deng

**Affiliations:** 1 Department of Transportation Engineering, College of Architectural Science and Engineering, Yangzhou University, Yangzhou, Jiangsu Province, China; 2 Institute of Urban Planning and Development, Yangzhou University, Yangzhou, Jiangsu Province, China; Southwest Jiaotong University, CHINA

## Abstract

Faced with the complex and diversified disturbances of abnormal events in high-speed railway, studying the characteristics of delay scenario parameters and related propagation influence laws plays a fundamental role in analyzing the applicability of operation plans, formulating train operation adjustments, and evaluating the operation decision plans, as well as providing practical data support for the establishment of theoretical models. Based on the mechanical analysis of the primary and knock-on delays, this paper combines the daily safety information data and timetable data, and takes into account the modified primary delays upon speed loss, by taking the high-speed railway network in the Yangtze River Delta region as an example. On this basis, this paper further studies the statistical distribution of primary delay duration, event location distribution, emergent measures distribution, occurrence time and event cause distribution, and other characteristics. Based on this research, the distribution characteristics of the influenced train number and the cumulative delay under kinds of disturbances have been discussed, where the effects of running redundancy, propagation rate, and related parameters on delay propagation have got quantitative analysis. Research shows that the number of abnormal event samples distributed in the sections is 1.53 times that of stations. Based on the Fuzzy C-Means clustering results, abnormal events in the section are more likely to propagate than abnormal events at stations. For trains experiencing primary delay, the relationship between the maximum cumulative train delay and primary delay mostly obey a power-law distribution and the values of R^2^ are all greater than 0.65, indicating a high correlation at the theoretical level.

## 1. Introduction

Recently, the high-speed railway (HSR) construction in China has developed rapidly. The national high-speed rail operating mileage has increased from 9,000 km in 2012 to 42,000 km in 2022. The construction of main HSR framework has been fully completed, and the construction of other corridors and general-speed trunk railways has been accelerated, resulting in the establishment of a railway network with a rational layout, extensive coverage, clear layers, and efficient configuration. During the operation of high-speed railways, they will inevitably face various complex and diverse abnormal events and disturbances, such as sudden failures, adverse weather conditions, and passenger behaviors. These disturbances often result in train delays and operational adjustments, significantly influencing the operational efficiency and service levels of the rail transport system [1, 2].

Existing research methods mainly focus on studying the train delay propagation law by analyzing the fastest catch-up speed of each train car based on the operating status of the preceding car and the time limit for train operation in the schedule [3]. Subsequently, the delay time of the rear car is calculated sequentially. Carey et al. utilized stochastic approximation to address the chain reaction caused by late trains and derived an approximate relationship between headway and chain delay [4]. Huisman et al. obtained the distribution of train running time by solving a system of linear differential equations [5]. The results obtained by these two methods are primarily achieved under ideal conditions and do not consider the influences of train and line performance, as well as the randomness and interdependence of contingencies in various scenarios. Burdett et al. proposed a sensitivity analysis to determine the influence of disturbances on the train schedule in order to assess its robustness [6]. Yuan et al. analyzed data from the Dutch railway network and found that late arrival delays and corresponding dwell delay at The Hague station scan generally be fitted to an exponential distribution and a normal distribution, respectively [7]. Yaghini et al. utilized a neural network model to study the delay propagation process of Italian railways and accurately predicted train delays [8]. Other scholars have studied the distribution of train delay duration and obtained train delay distribution curves suitable for different railway lines [9, 10]. Xu et al. drew a distribution curve of high-speed train delays based on actual train operation data, but their research did not establish a relevant model for the cause—the primary delay duration [11]. Yin et al. established a station delay propagation model based on the SIR model to analyze the delay propagation mechanism at station [12]. Huang et al. used the operation data of trains on the Wuhan-Guangzhou HSR in China to establish a probability distribution model for the number of delayed trains for various types of faults [13]. Hou et al. also established a SIS infectious disease dynamics model of train delay propagation based on this data, and explored the final trend of delay propagation within the system based on stationarity theory [14]. Hu et al. studied the delay propagation process and propagation laws of various types of train sets operating in the section based on probability theory and stochastic process theory [15]. Yang et al. developed a train delay propagation simulation system to test the dynamic performance of train operation [16]. In addition, some studies have analyzed the influence of delay propagation across the railroad network. For example, Zhu et al. explored the relationship between late trains and technical failures. The technical failures of the railroad system were classified into technical failures at stations, track sections, and on trains. The influence of these technical failures on train operations was analyzed and formulated to evaluate the performance of the railroad system under random disturbances [17]. Goverde et al. proposed a model and algorithm to compute the propagation of primary delays over a periodic train schedule, by modeling the railroad system as a linear system. The propagation of train delay was computed through time event diagrams using the method of graph algebra, and the delay propagation properties and convergence of the algorithm were analyzed [18]. Bueker used activity diagrams to represent the delay propagation, where a suitable class of distribution functions was employed to describe the delays as random variables, and total delays can be efficiently obtained by calculating the cumulative distribution function [19]. Table 1 compares and summarizes the research methods, research objects, research scenarios, and research purposes of studies related to the propagation of high-speed railway train delays.

**Table 1:** Summary table of analysis methods for train delay propagation patterns at home and abroad.

Ref	Method	Focus	Scenario	Purpose
Carey et al. [4]	• Stochastic approximation • Simulation analysis	• Train delay	• Medium disruption	• Train planning and dispatching • Train timetable planning
Huisman et al. [5]	• Mathematical modelling	• Train delay	• Small disturbance • Medium disruption	• Railway planning
Burdett et al. [6]	• Sensitivity analysis	• Train delay	Small disturbance	• Train timetable planning
Yuan et al. [7]	• Mathematical modeling	• Train station delay	• Small disturbance	• Train delay prediction
Yaghini et al. [8]	• Mathematical modeling	• Train delay	• Small disturbance • Medium disruption	• Train delay prediction
Yin et al. [12]	• Mathematical modelling • Simulation analysis	• Train station delay	• Small disturbance • Large-scale delay	• Emergency dispatching
Hu et al. [15]	• Theoretical research	• Dispatching section delay	• Small disturbance	• Railway transportation management
Zhu et al. [17]	• Mathematical modelling • Simulation analysis	• Train delay	• Small disturbance • Medium disruption	• Train delay prediction
Goverde et al. [18]	• Mathematical modeling	• Train delay	• Large-scale delay	• Emergency dispatching

In order to address abnormal event disturbances in high-speed railway operations and optimize the effectiveness of train operation adjustment models and operation plans, it is crucial to conduct in-depth research on the parameter characteristics of delay scenarios and their propagation influence rules. By analyzing the parameter characteristics and propagation influence rules of delay scenarios, this paper can provide fundamental guidance and empirical data support for adjusting high-speed railway operation plans. This can enhance the safe and efficient operation of high-speed railway transportation systems, in order to promote the sustainable operational management of railway transportation organizations.

The research content of this paper is as follows. Section 2 details the mechanism of high-speed railway train delays. Section 3 introduces data collection and parameter feature analysis of high-speed railway train delay scenarios, considering the primary delay correction of speed loss. Section 4 studies the influence analysis of high-speed railway train delays and related strategy suggestions. Finally, Section 5 concludes the paper with major contributions and innovations.

## 2. Train delay mechanisms in high-speed railways

### 2.1. The primary train delays

Due to the diversity and randomness of abnormal events in high-speed railroads, the disposal measures vary depending on the event types and disturbance levels. The primary train delay is majorly associated with the event types and the measures implemented to reorganize the train schedule. It should be noted that, even without the disturbance of abnormal events, high-speed railroad trains may still experience random minor schedule deviations during operation. These deviations are not within the scope of the research discussed in this paper.

**Fig 1:**
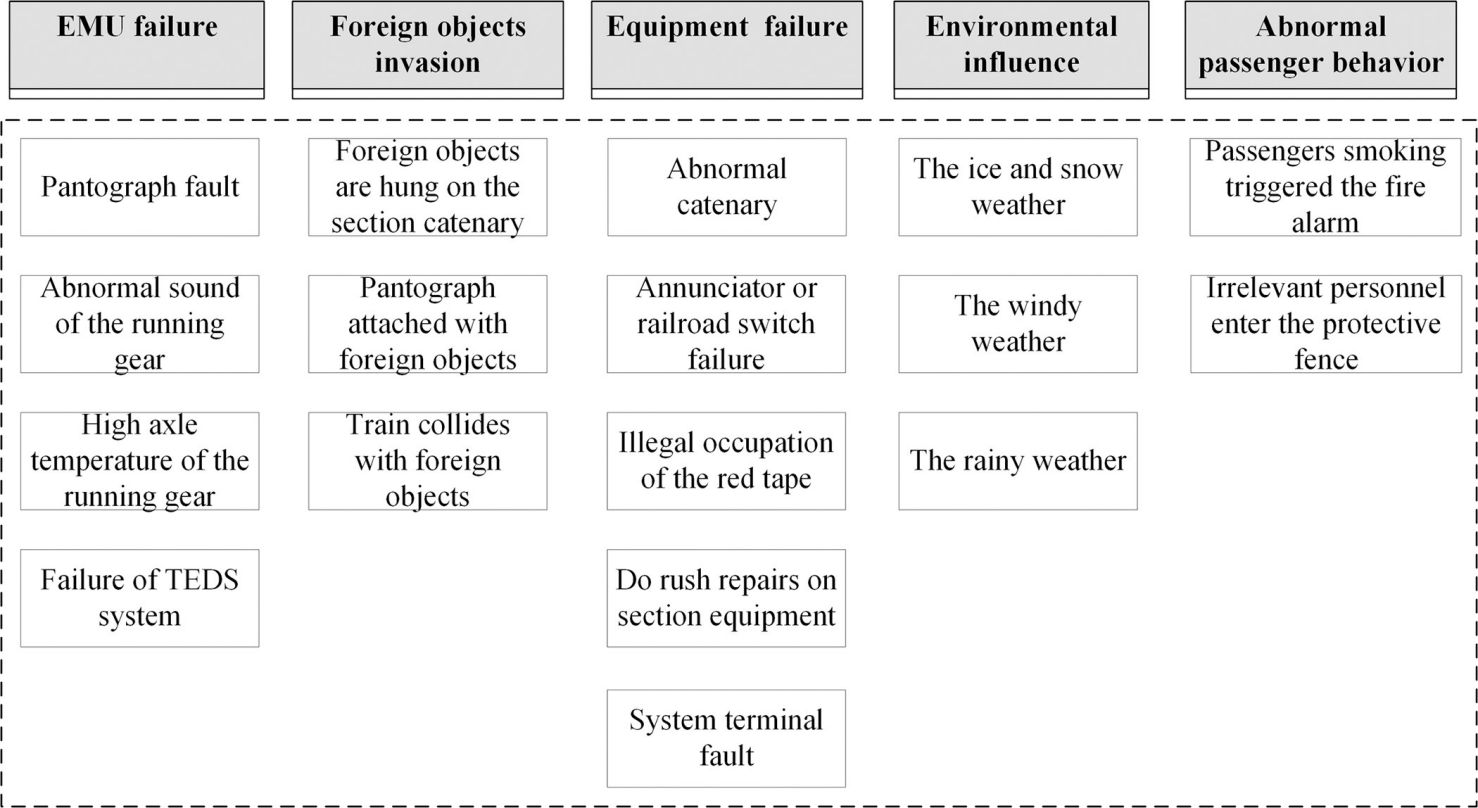
Typical abnormal events and causes of high-speed railway.

**Fig 2:**
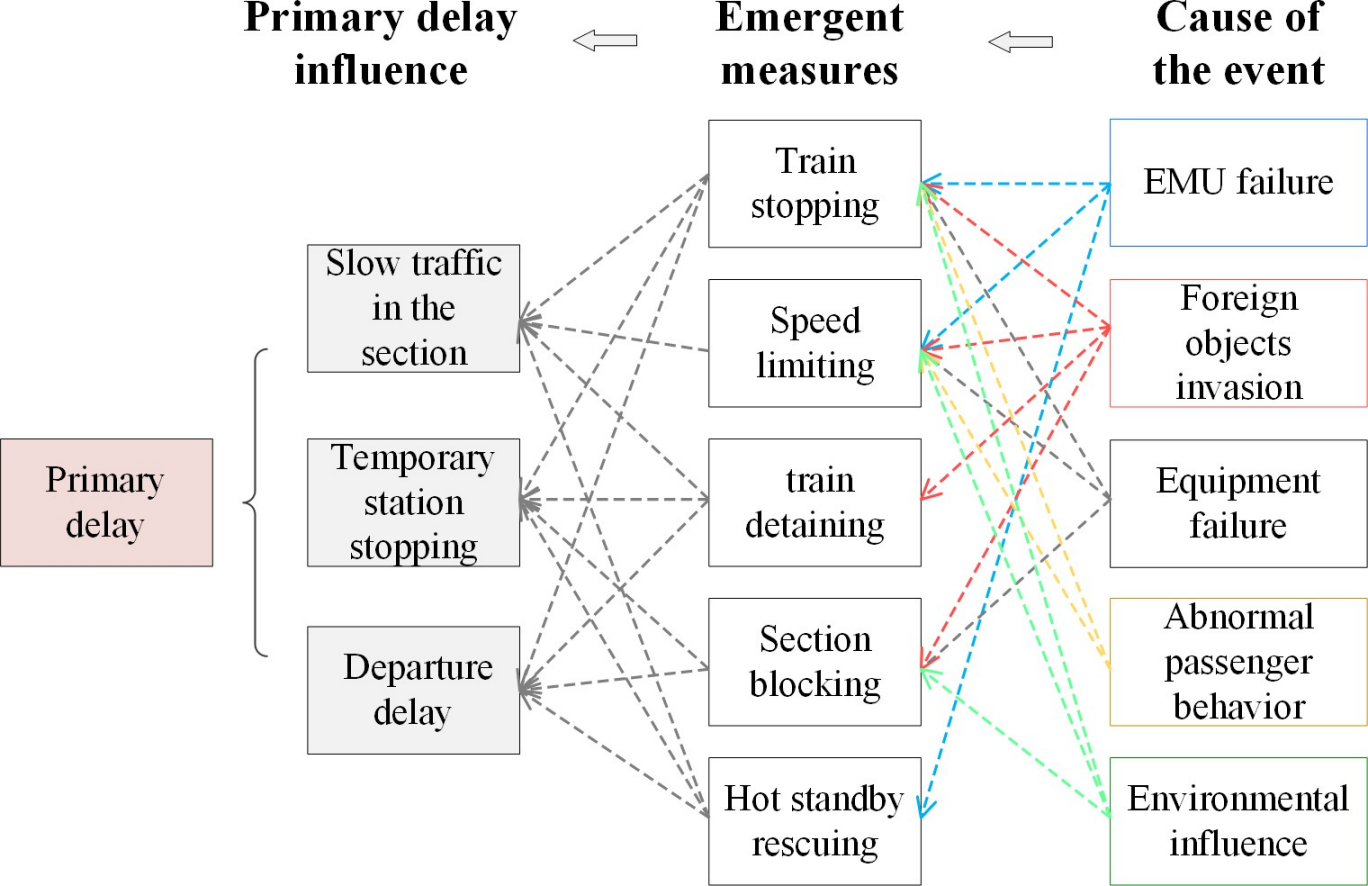
Network structure of basic causes of primary train delay.

For the recoverable abnormal event disturbance scenario studied in this paper, Fig 1 proposes typical event causes corresponding to 5 common event types. Through correlation analysis of Fig 1, a specific cause relationship network can be teased out. As shown in Fig 2, under the main abnormal event cause types of EMU failure, foreign objects invasion, equipment failure, passenger behavior, environmental influence, and abnormal passenger behavior, according to the characteristics of the disturbance scenario and the severity of the event, emergent measures such as train stopping, speed limiting, train detaining, section blocking, hot standby rescuing, and other related measures should be taken. Different emergent measures will have different types of primary delay influences on the first influenced train, mainly including slowdowns in the section, temporary station stopping, and departure delay (including original departure delay and departure delay due to overtime station stopping).

It can be analyzed that there are differences in the normal emergent measures under various causes of events. For instance, EMU failures often result in stopping and checking, speed limiting, or hot standby rescuing. On the other hand, equipment failures as well as external environmental influences, typically lead to train stopping, speed limiting or section blocking. At the same time, because the abnormal event occurs at both stations and sections, there are variations in the primary delay effects of different emergent measures. For instance, speed limiting primarily results in additional section running time, while section blocking mainly causes temporary station stopping and departure delay.

### 2.2. Knock-on train delays

**Fig 3:**
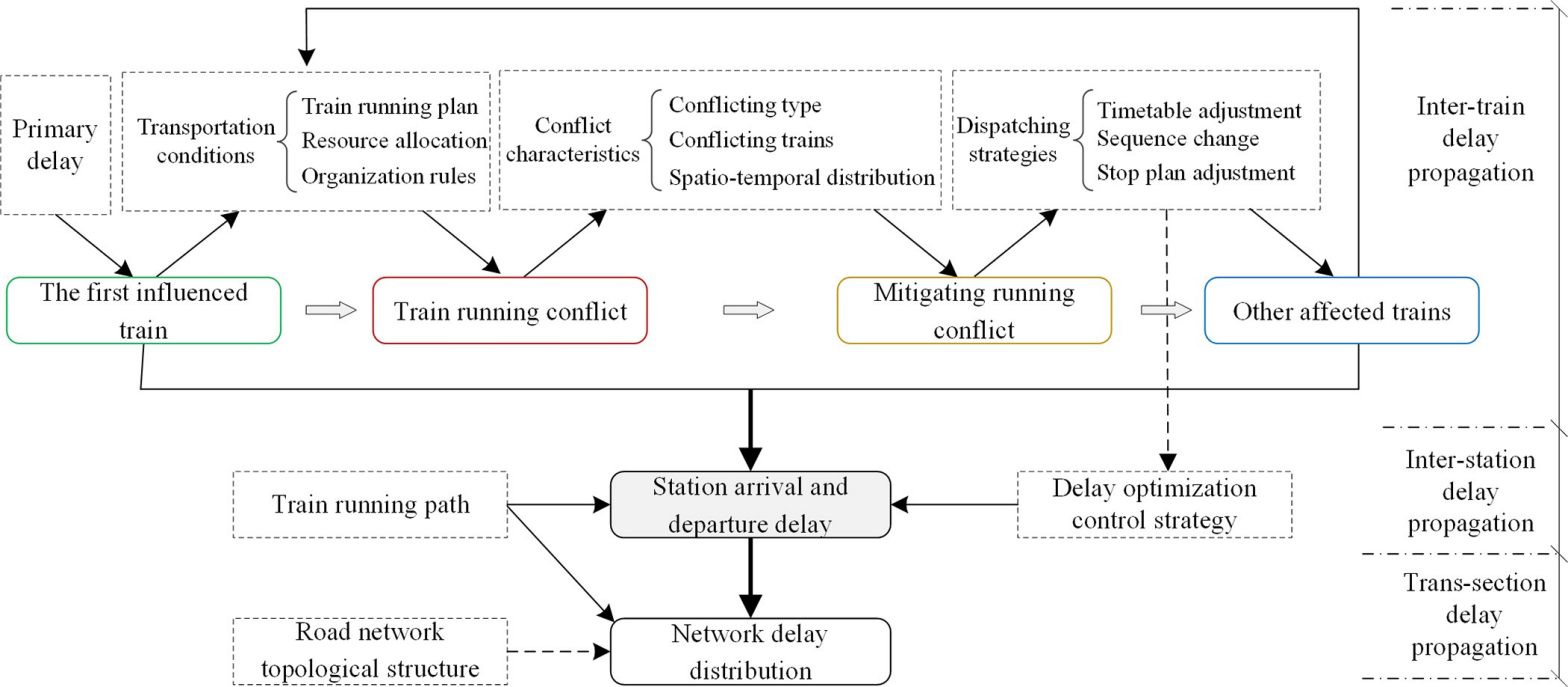
The generation and propagation mechanism of knock-on train delay.

The process of knock-on delay generation is equivalent to the process of delay propagation. By analyzing the characteristics of train delay scenarios, this paper divides delay propagation into inter-train delay propagation, inter-station delay propagation, and trans-section delay propagation, as shown in Fig 3.

The delay mechanism for each propagation stage is as follows:

- Inter-train delay propagation

Inter-train delay refers to the delay in train operations caused by resolving conflicts between two adjacent trains. Due to the mitigation strategy, the operation plans of other affected trains will be adjusted. There is a need to cycle through conflict detection and conflict mitigation until there are no traffic conflicts on the road network. In this closed-loop control process, the influenced trains generate a chain of delays formed by inter-train delay propagation.

- Inter-station delay propagation

Inter-station delay propagation refers to the process of arrival and departure delays affecting trains at stations in the forward direction of the operating path. Trains that have been delayed can be hurry through utilizing subsequent intervals, compressing station stopping time, and implementing other measures to strive for punctuality. As a result, station arrival and departure delays are progressively decreasing. Additionally, some trains may bypass high-traffic stations, adjust their stopping times, or implement other strategies, leading to dynamic fluctuations in station arrival and departure delays.

- Trans-section delay propagation

Due to the variability of high-speed railway train running paths and the complexity of the road network topology, networked train delay propagation across scheduling segments will occur within a certain range when facing abnormal event perturbations, such as heavy primary delays or abnormal events occurring within the network hub stations and adjacent sections.

## 3. Data collection and characterization of HSR delay scenarios

### 3.1. Data collecting and filtering

#### (1) Data source

**Fig 4:**
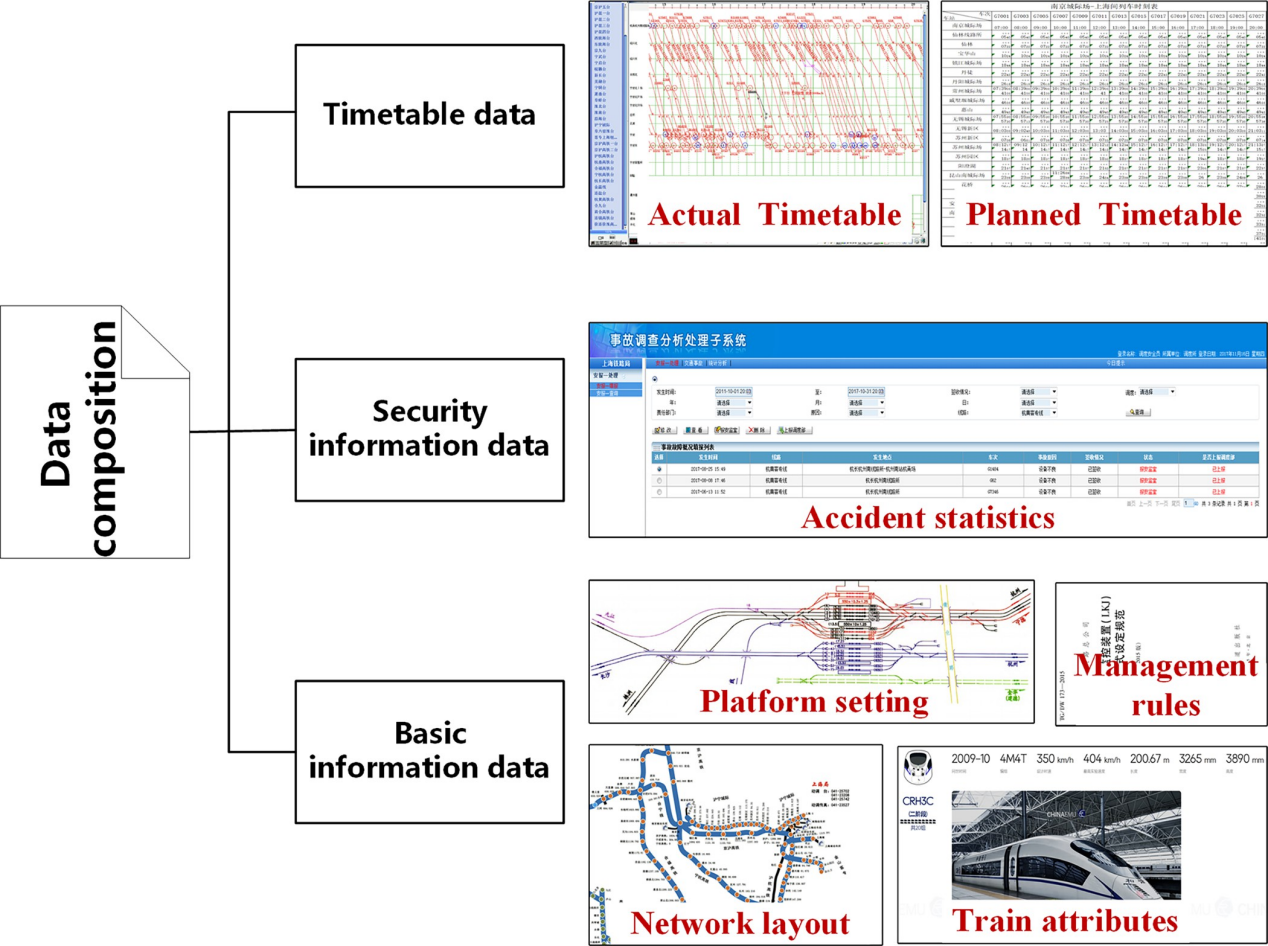
Data source.

In this paper, the basic information data, daily safety information data and timetable data of high-speed railways from Jun 2019 to Nov 2020 serve as the data source for the study, as shown in Fig 4. Among them, the safety information data records the information about the lines, train numbers, locations, disposal processes, and actual impacts of abnormal events in the study area in text form, mainly for subsequent extraction of characteristic attribute parameters of abnormal events. The timetable data is derived from the relevant dispatching documents within the corresponding data period, detailing the train operation plans for each dispatching section within the jurisdiction. It is primarily used to extract the train transportation conditions at the spatial and temporal locations of abnormal events, forming a supplement to the characteristic parameters of the delay scenarios caused by abnormal events. The basic information data mainly includes national regulations, train standards, and information on railway network layout and platform settings of relevant railway departments. It is mainly used for supplementing and standardizing subsequent data processing.

#### (2) Data filtering

The research object of this paper is the study of high-speed railway train operation delays and their propagation influence characteristics, which need to be preliminary screened according to the research needs to clarify the research scope. The basic principles of railway lines selecting are as follows:

- Partial railway lines are outside the scope of the study due to the small number of high-speed trains, low design speed, and being a mixed passenger and freight railway, and some lines that are still in the early operation stage is not considered due to their low traffic volume.
- Partial connecting lines and detour lines are merged with adjacent lines, and the relevant parameters are taken according to the standard values of adjacent lines.

Meanwhile, the basic parameters involved in data correction, such as railway lines and train models, are shown in Table 2.

**Table 2:** Basic train information of representative HSR lines within current network.

Railway Line	Operating section	Design speed (km/h)	Main train models	Top speed (km/h)	Train control system
Hangzhou-Shenzhen Railway	Hangzhou East-Ningbo	350	CRH380BL	300	CTCS-3
	Ningbo- Wenzhou South	250	CRH1B\E, CRH2A\B\C\E, CRH380CL, CR400BF	250	CTCS-2
	Wenzhou South- Cangnan	250	CRH1B\E, CRH2A\B\C\E, CRH380CL, CR400BF	200	CTCS-2
Shanghai-Nanjing Intercity Railway	Shanghai- Nanjing	300 (locally 350)	CRH3\2C, CRH380B\D, CR400AF\BF	300	CTCS-3
Beijing–Shanghai High-speed Railway	Shanghai Hongqiao- Xuzhou East	380	CR400AF\BF	350	CTCS-3
			CRH380A(L)\B(L)\C(L)\D, CRH2C	300	
			CRH2A\2E	250	

To facilitate subsequent analysis, in addition to textual information, the following nine attribute parameters can be extracted: train number, location of the railway line, time of occurrence, cause of the event, train number, event location, emergent measures, event disposal time, number of influenced trains, direction of the influence.

### 3.2. The primary delay correction considering speed loss

By combining the number of trains, railway line sections, and emergent measures, we calculate the possible additional running time from starting and stopping, the lost time from speed reduction, and correct the event disposal time to determine the primary delay of the corresponding trips, where the time lost due to speed reduction includes the additional running time due to acceleration and deceleration, as well as the time lost due to the enforcement of speed limit strategy.

#### 3.2.1. Average deceleration

**Fig 5:**
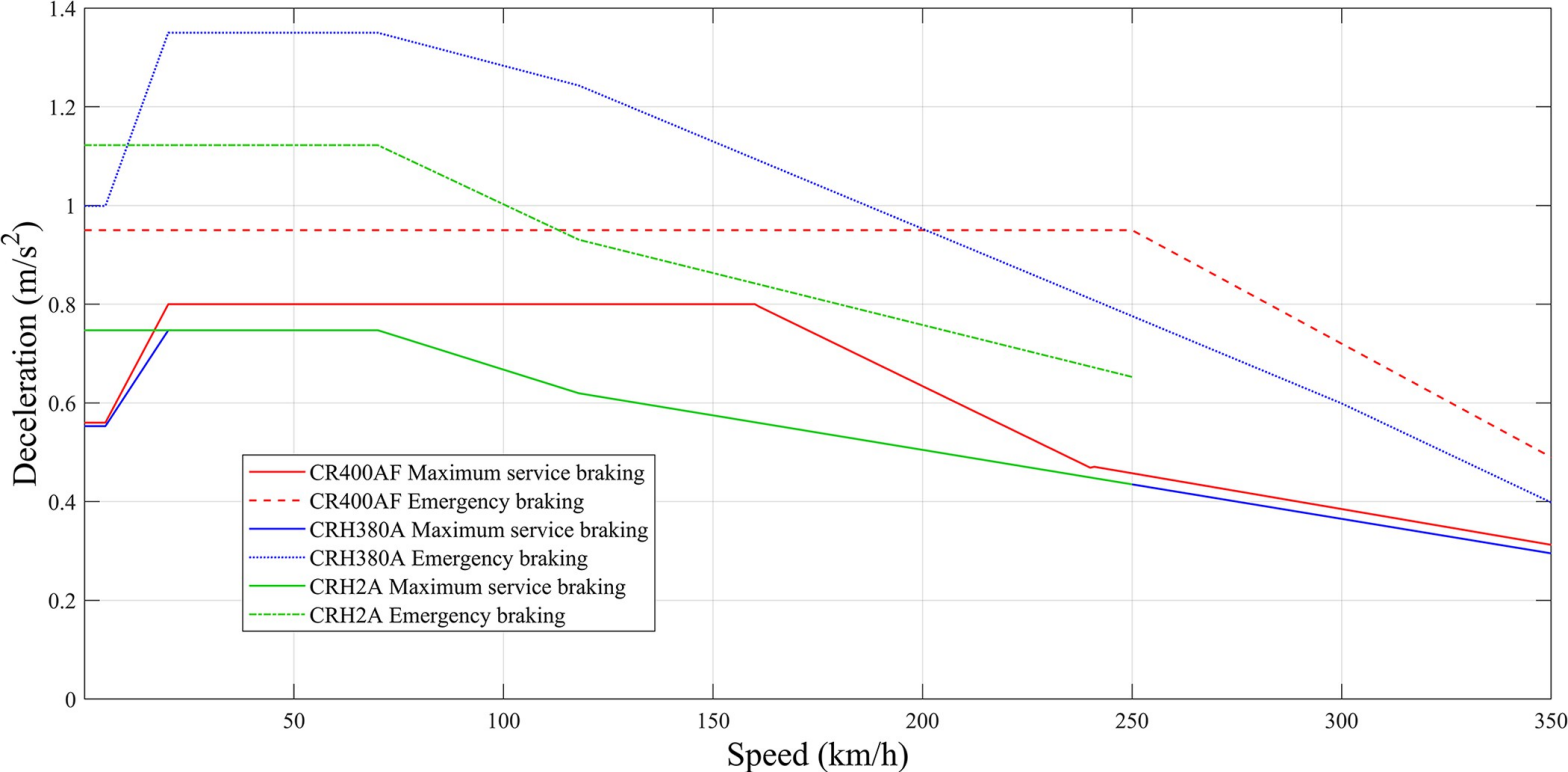
Speed-deceleration curves of two representative trains under two braking modes.

Due to the complexity of the rolling stock types on various railway lines, representative train models from different speed classes are selected. The average deceleration of each representative model is then determined separately as they decelerate from different primary speeds to various speed limits using different braking modes. For the speed limit dispatching order, the maximum normal braking mode is generally adopted, while for the stopping order, it needs to be calculated separately based on the actual recorded information. According to the braking parameter calculation method specified in the *Specification of settings for control mode of train monitoring device* (*2015 Edition*) [20], the braking curves of representative models under each operating speed class for two braking modes are illustrated in Fig 5.

As shown in Fig 5, since the braking curves under different braking modes are piecewise functions, the center of gravity method can be used to determine the average deceleration of each train model under different braking modes when braking from different operating speeds (*v*_*0*_) to the target speed limit (*v*_*l*_). Letting *f*(*v*) denotes the braking function, the average deceleration can be calculated by:

$${\bar{a}}_{D}=\frac{\int_{v_{l}}^{v_{0}}f(v)dv}{v_{0}-v_{l}}$$ (1)

When the velocity interval [*v*_*l*_, *v*_*0*_] corresponding to the deceleration are on the same line segment, *f*(*v*) is either a constant or a linear function of the velocity *v*. The average deceleration is the constant corresponding to the braking start and stop velocities. When *f*(*v*) is a constant, the average deceleration is the corresponding constant; when *f*(*v*) is a linear function of *v*, the average deceleration is the average value of deceleration corresponding to the braking starting and stopping velocities, as indicated in Eq (2) and Eq (3):

$$f(v)=c_{1}∙v+c_{2}$$ (2)

$${\bar{a}}_{D}=\frac{\int_{v_{l}}^{v_{0}}{(c}_{1}∙v+c_{2})dv}{v_{0}-v_{l}}=\frac{f(v_{0})+f(v_{l})}{2}$$ (3)

When the deceleration rates within the interval of [*v*_*l*_, *v*_*0*_] are not on the same line segment, i.e., they span different intervals of the deceleration piecewise function. Taking the spanning of two deceleration intervals as an example, the two functions involved are *f*_*1*_(*v*) and *f*_*2*_(*v*), and the dividing velocity point is *v*_*s*_, then we have:

$${\bar{a}}_{D}=\frac{\int_{v_{l}}^{v_{s}}f_{1}(v)dv+\int_{v_{s}}^{v_{0}}f_{2}(v)dv}{{(v}_{0}-v_{s})+(v_{s}-v_{l})}=\frac{{\bar{a}}_{D1}∙(v_{s}-v_{l})+{\bar{a}}_{D2}∙(v_{0}-v_{s})}{{(v}_{0}-v_{s})+(v_{s}-v_{l})}$$ (4)

Let *v*_*s*_−*v*_*l*_ = Δ*v*_1_, and *v*_0_−*v*_*s*_ = Δ*v*_2_, which can be obtained by substituting into the above equation:

$${\bar{a}}_{D}=\frac{{\bar{a}}_{D1}\Delta v_{1}+{\bar{a}}_{D2}\Delta v_{2}}{\Delta v_{1}+\Delta v_{2}}$$ (5)

By analogy, when the velocity difference spans n intervals of the deceleration segmentation function, the average deceleration is obtained:

$${\bar{a}}_{D}=\frac{\sum_{i=1}^{n}{\bar{a}}_{Di}\Delta v_{i}}{\sum_{i=1}^{n}\Delta v_{i}}$$ (6)

#### 3.2.2. Time of speed loss

**Fig 6:**
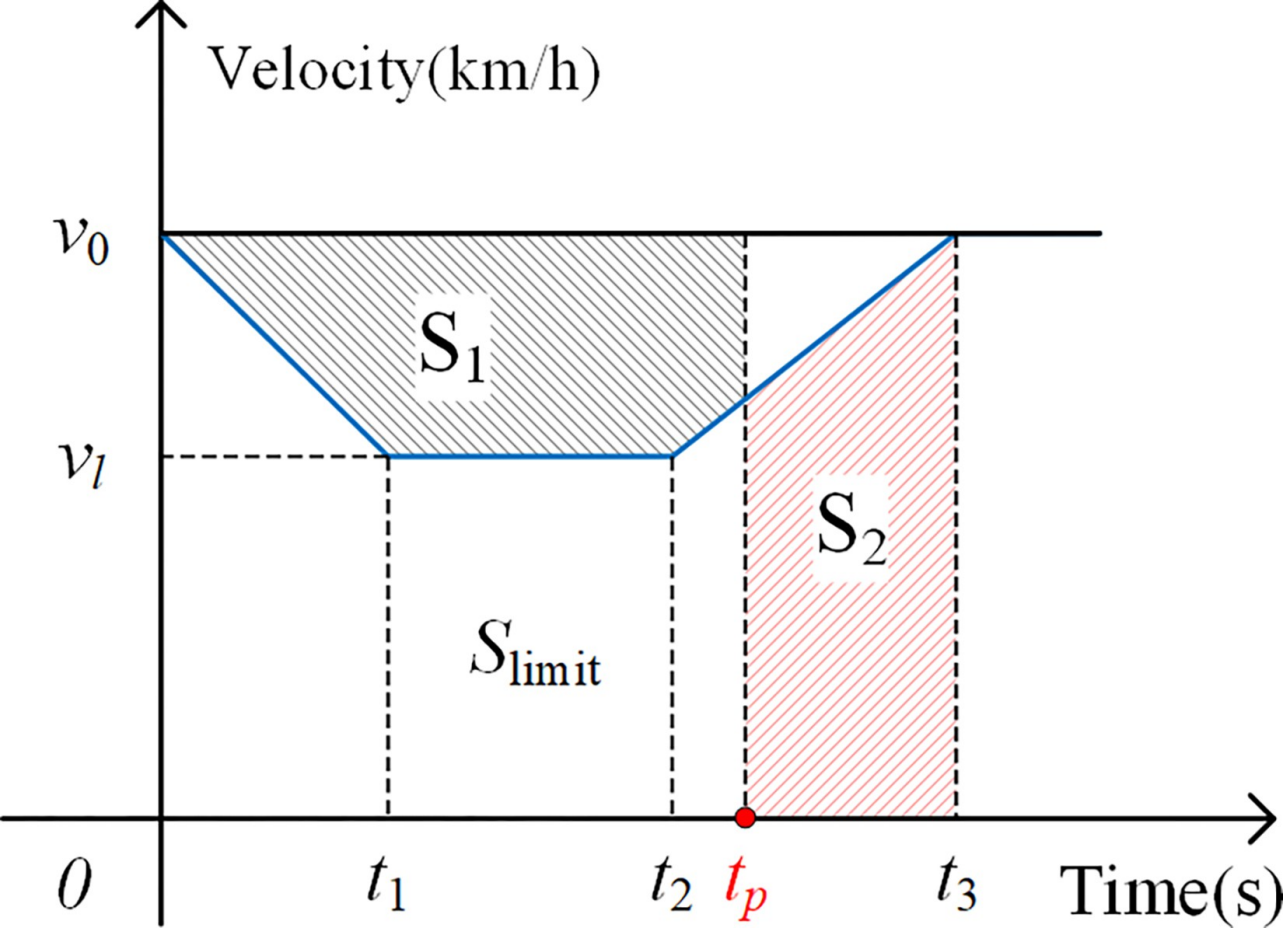
Schematic diagram of speed loss time under speed restriction measures (S1 = S2).

In the definition of speed loss time, it is illustrated in Fig 6. Let the starting moment of braking be 0, the time consumption of decelerating from *v*_0_ to *v*_*l*_ is *t*_*1*_. The speed limit operation is maintained during the period from *t*_*1*_ to *t*_*2*_. The total time consumption required from the start of braking to the restoration of the normal speed is *t*_*3*_. The actual train running distance during the time period *t*_*3*_ is *S*_*limit*_. The time required for the train to complete the distance *S*_*limit*_ at the primary normal speed is recorded as *t*_*p*_. The difference between *t*_*3*_ and *t*_*p*_ is the speed loss time under the influence of speed limiting measures. Additionally, for the speed loss time generated by the station stopping and interval stopping measures, both can be regarded as the speed reduction loss time in the case that the target value of the speed limit is 0, but with additional stopping loss time.

According to the relevant parameter settings, the train travel distance under the speed limit strategy is calculated according to Eq (7):

$$S_{limit}=\frac{{(v}_{0}+v_{l})∙t_{1}}{3.6\times 2}+\frac{v_{l}∙(t_{2}-t_{1})}{3.6}+\frac{{(v}_{0}+v_{l})∙(t_{3}-t_{2})}{3.6\times 2}$$ (7)

where:

$$t_{1}=\frac{v_{0}-v_{l}}{3.6\times {\bar{a}}_{D}},\;t_{2}-t_{1}=\Delta t_{L},\;t_{3}-t_{2}=\frac{v_{0}-v_{l}}{3.6\times {\bar{a}}_{A}}$$ (8)

where ${\bar{a}}_{D}$ and ${\bar{a}}_{A}$ are the average deceleration and average acceleration, respectively, m/s^2^. The average deceleration is calculated by applying Eq (6) with the braking curve function of the corresponding train type; the average acceleration is determined based on the starting acceleration in the performance parameters of the corresponding train type. Specifically, 0.36 m/s^2^ is used for the CRH2A and the similar types, 0.39 m/s^2^ for the CRH380 series, and 0.53 m/s^2^ for the CR400 series.

substituting into Eq (8) there is:

$$S_{limit}=\frac{{v_{0}}^{2}-{v_{l}}^{2}}{25.92\times {\bar{a}}_{D}}+\frac{v_{l}∙\Delta t_{L}}{3.6}+\frac{{v_{0}}^{2}-{v_{l}}^{2}}{25.92\times {\bar{a}}_{A}}$$ (9)

and then we have:

$$t_{p}=\frac{S_{limit}}{v_{0}∕3.6}=\frac{1}{v_{0}}(\frac{{v_{0}}^{2}-{v_{l}}^{2}}{7.2\times {\bar{a}}_{D}}+v_{l}∙\Delta t_{L}+\frac{{v_{0}}^{2}-{v_{l}}^{2}}{7.2\times {\bar{a}}_{A}})$$ (10)

$$t_{3}=\frac{v_{0-}v_{l}}{3.6\times {\bar{a}}_{D}}+\Delta t_{L}+\frac{v_{0-}v_{l}}{3.6\times {\bar{a}}_{A}}$$ (11)

therefore, the time loss under speed-limited operation can be obtained as:

$$t_{loss}=t_{3}-t_{p}$$ (12)

The description of speed limits in the daily safety information text in China includes both temporal and spatial speed limits: one is "operating at a speed limit of ×× km/h for ×× min"; the other is "operating at a speed limit of ×× km/h for ×× kilometers before and after the location of the accident". For the former, the relevant parameters are calculated by substituting them into the aforementioned formula; for the latter, the second term in the Eq (9) for calculating the speed limit running distance *S*_*limit*_ is the speed limit mileage, and the speed limit running time Δ*t*_*L*_ needs to be calculated based on the speed limit running distance and the speed limit. Based on this, the speed loss time under commonly used strategies can be calculated (as shown in Table 3), and the event resolution time is adjusted to the primary delay accordingly.

**Table 3:** List of speed loss time under common emergent strategies (min).

Train type	Primary velocity (km/h)	5min speed limit value (km/h)				4km speed limit value (km/h)				Temporary stopping	
		200	160	120	80	200	160	120	80	NB^1^	EB^2^
**CR400AF\BF**	350	2.74	3.59	4.53	5.50	1.11	1.69	2.56	3.96	2.76	2.44
	300	1.96	2.87	3.86	4.93	0.70	1.24	2.06	3.46	2.31	2.06
	250	1.09	2.06	3.12	4.28	0.33	0.80	1.56	2.92	1.89	1.70
	200	\	1.06	2.24	3.53	0.00	0.36	1.04	2.33	1.48	1.36
**CRH380 and CRH2C**	300	2.04	3.05	4.15	5.31	0.78	1.42	2.35	3.85	2.99	2.46
	250	1.11	2.14	3.30	4.55	0.35	0.88	1.74	3.19	2.44	2.01
	200	\	1.08	2.32	3.70	\	0.38	1.12	2.50	1.92	1.59
**Other CRH1 and CRH2**	250	1.11	2.16	3.33	4.61	0.35	0.90	1.77	3.25	2.56	2.13
	200	\	1.09	2.34	3.73	\	0.39	1.14	2.53	2.02	1.68

^1,2^ NB in the table indicates the use of maximum service braking mode, and EB indicates the use of emergent braking mode.

#### 3.2.3. Sectional distribution of train number

Since the number of pairs of trains running at different sections on the same railway line varies, and the frequency of trains running at different times is not consistent, it is essential to analyze the spatial and temporal distribution characteristics of the number of train pairs operating in each scheduling section using timetable data. This analysis helps in understanding the train operation status during the occurrence of abnormal events. By integrating this information with traffic safety data, a set of scenario characteristic parameters for the abnormal event can be established.

*(1) Extracting the number of full-day trains in the section*. According to the operating schedule data, the number of full-day train pairs in each section on various scheduling sections is extracted. Taking the Beijing-Shanghai high-speed railway East Xuzhou Station as an example, the recorded format of representative train operation time is shown in Table 4.

**Table 4:** Data format of representative train timetable of East Xuzhou Railway Station.

Station	Train number	G1	G33	G1665	G207
East Xuzhou Railway Station	Arrival time	. . .	12:53:26		22:45
	Departure time	11:18:35	12:57:09	07:20	--
**Train type**		Pass	Stop and pass	Original departure	Final arrival

The number of trains in the operating section ahead of the current station is the sum of the corresponding number of passing trains, stopping and passing trains, and original departure trains. In other words, it is the total number of trains of each type at the station minus the number of terminating trains.

*(2) Extraction of the time distribution of the number of running trains in the section*. Considering the delay propagation scope of abnormal events and the running time consumption of trains in the dispatching section, the time distribution of the number of train pairs on the middle section of each line is counted by 2-hour intervals. The time distribution of the number of train pairs on typical railway line sections is shown in Table 5. Due to the longer operating mileage of the railway lines managed by the Shanghai Railway Bureau, the *Beijing-Shanghai* HSR is divided into *Xuzhou-Nanjing* section and *Nanjing-Shanghai* section, the *Shanghai-Kunming* HSR is divided into *Shanghai-Hangzhou* section and *Hangzhou-Jiangshan* section, and the *Hangzhou-Shenzhen* HSR is divided into *Hangzhou-Ningbo* section and *Ningbo-Wenzhou* section.

**Table 5:** Data format of representative train timetable of East Xuzhou Railway Station.

Period of time	Beijing-Shanghai HSR (Nanjing-Shanghai)	Nanjing-Hangzhou HSR	Nanjing-Shanghai HSR	Hefei-Fuzhou HSR (Hefei-Huangshan)	Shanghai-Kunming HSR (Shanghai-Hangzhou)
**0:00~2:00**	0.38%	0.00%	0.00%	0.00%	2.72%
**2:00~4:00**	0.38%	0.00%	0.00%	0.00%	2.72%
**4:00~6:00**	0.57%	2.00%	0.00%	1.96%	1.36%
**6:00~8:00**	3.64%	8.00%	7.56%	9.80%	10.88%
**8:00~10:00**	8.81%	16.00%	12.61%	15.69%	11.56%
**10:00~12:00**	12.26%	16.00%	12.18%	13.73%	12.93%
**12:00~14:00**	13.22%	14.00%	12.61%	11.76%	12.93%
**14:00~16:00**	12.64%	13.00%	13.45%	19.61%	10.88%
**16:00~18:00**	13.03%	13.00%	12.61%	13.73%	13.61%
**18:00~20:00**	14.37%	12.00%	13.03%	7.84%	10.88%
**20:00~22:00**	11.88%	6.00%	15.13%	5.88%	8.84%
**22:00~24:00**	8.81%	0.00%	0.84%	0.00%	0.68%

### 3.3. Characterization of delay scenario parameters

Taking the 11 high-speed railway lines under the control of the Shanghai Railway Bureau in the Yangtze River Delta region, such as the *Beijing-Shanghai* HSR, *Shanghai-Nanjing* intercity Railway, *Nanjing-Anqing* HSR, and *Hangzhou-Shenzhen* HSR, which operate trains in moving groups, as the focus, a total of 501 valid abnormal event data within the research scope were collected. The original train number information and event disposal time in the attribute parameter of 3.1 are unified and corrected into the primary delay by combining the additional time calculation. Additionally, the number of pairs of trains running throughout the day in Section 3.2.3 is also combined with the time distribution of the number of trains running in the section’s interval to transform into the service frequency of the incident railway line’s section. Based on this, scenario characterization is conducted.

#### (1) Distribution of the primary delay lengths

**Fig 7:**
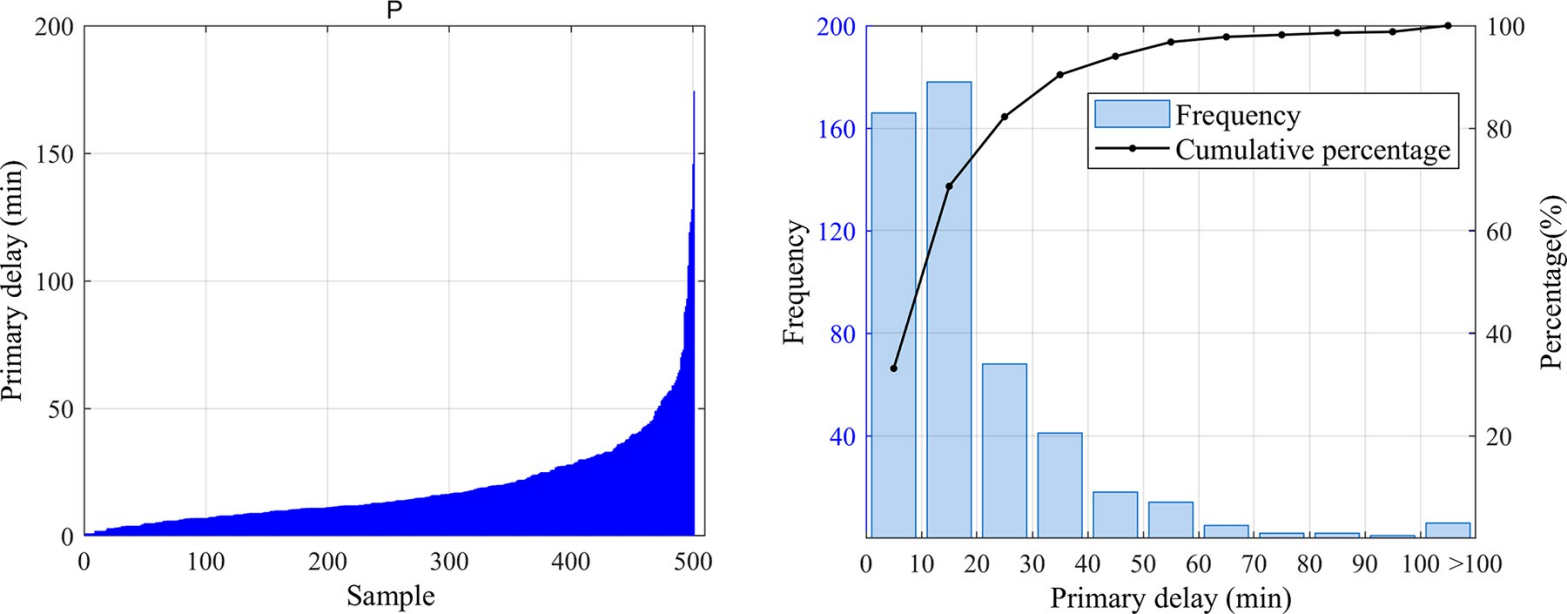
Primary delay distribution of abnormal events. (a) Sorted primary delay distribution; (b) Frequency distribution of primary delays.

As shown in Fig 7, the primary delay length distribution of abnormal events in high-speed railways varies from 1 to 175 minutes based on the processed sample data.

#### (2) Distribution of event location

For the distribution of abnormal event positions, the analysis primarily focuses on combining the railway line with the location of occurrence to examine abnormal events in sections and stations. According to relevant statistics show that, among the current 501 samples of abnormal events on high-speed railways, there are 303 abnormal events distributed in sections and 198 abnormal events distributed at stations. Among the section abnormal events, the *Beijing-Shanghai* HSR, *Shanghai-Nanjing* intercity, and *Hangzhou-Shenzhen* HSR have a higher proportion of occurrences, accounting for 29.70%, 15.84%, and 13.20% respectively. At the station level, the *Beijing-Shanghai* HSR, *Shanghai-Kunming* HSR, and *Hangzhou-Shenzhen* HSR also have a higher proportion of event occurrences.

#### (3) Distribution of emergent measures

**Fig 8:**
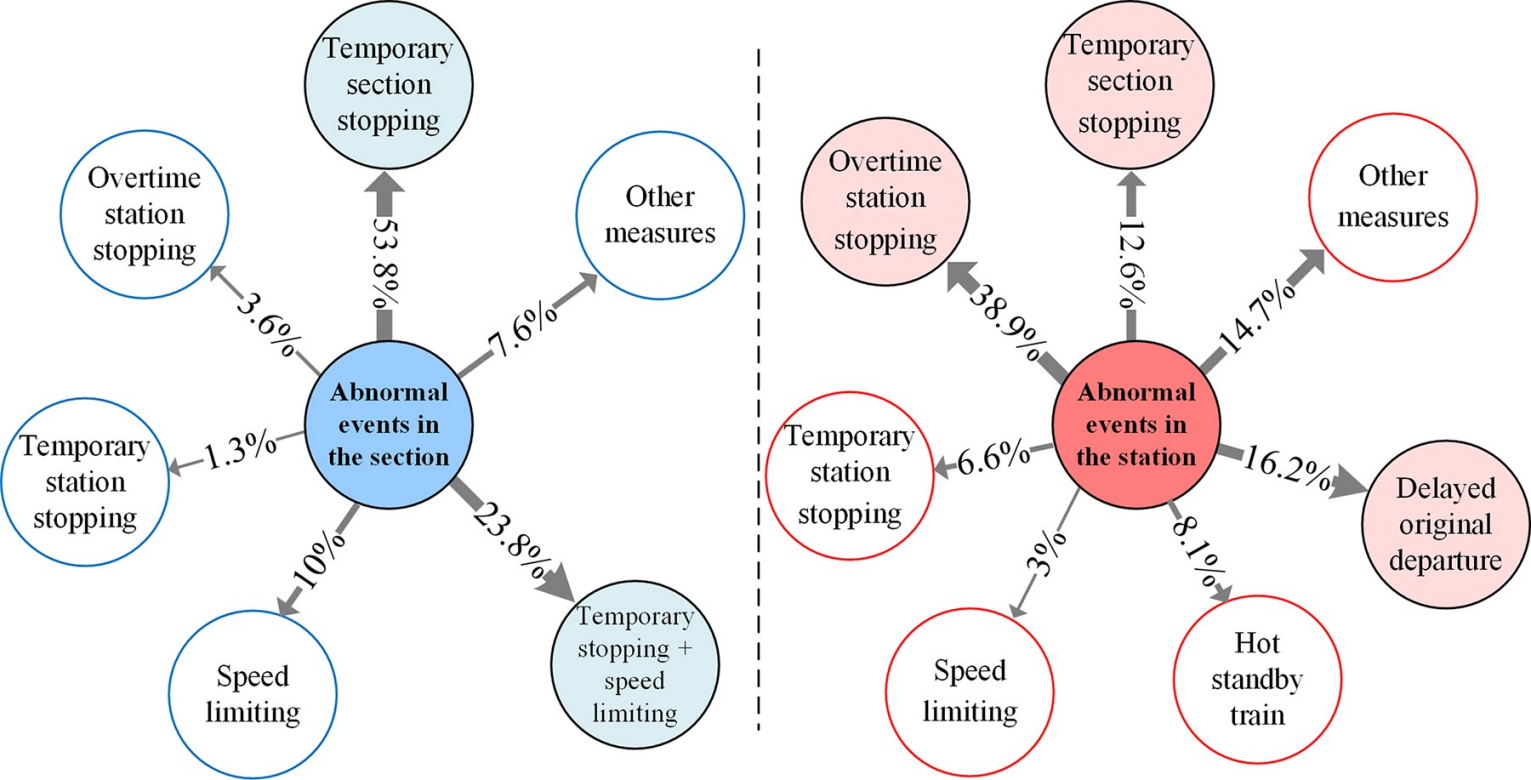
Distribution of emergent measures for sections and stations under abnormal events.

The distribution of normal disposal strategies for abnormal events in sections and stations is shown in Fig 8. For abnormal events in the sections, temporary section stopping and combined temporary section stopping and speed limiting are mainly used, while the measure of speed limiting in the sections alone is less normal. In rare cases, measures such as overtime station stopping or temporary station stopping will be taken. For abnormal events at stations, the main measures are overtime station stopping, delayed original departure and temporary section stopping. Among them, delayed original departure is different from overtime station stopping. The trains of delayed original departure are mostly located in the train depots, waiting for the station entrance operation.

#### (4) Distribution of occurrence time and cause of event

**Fig 9:**
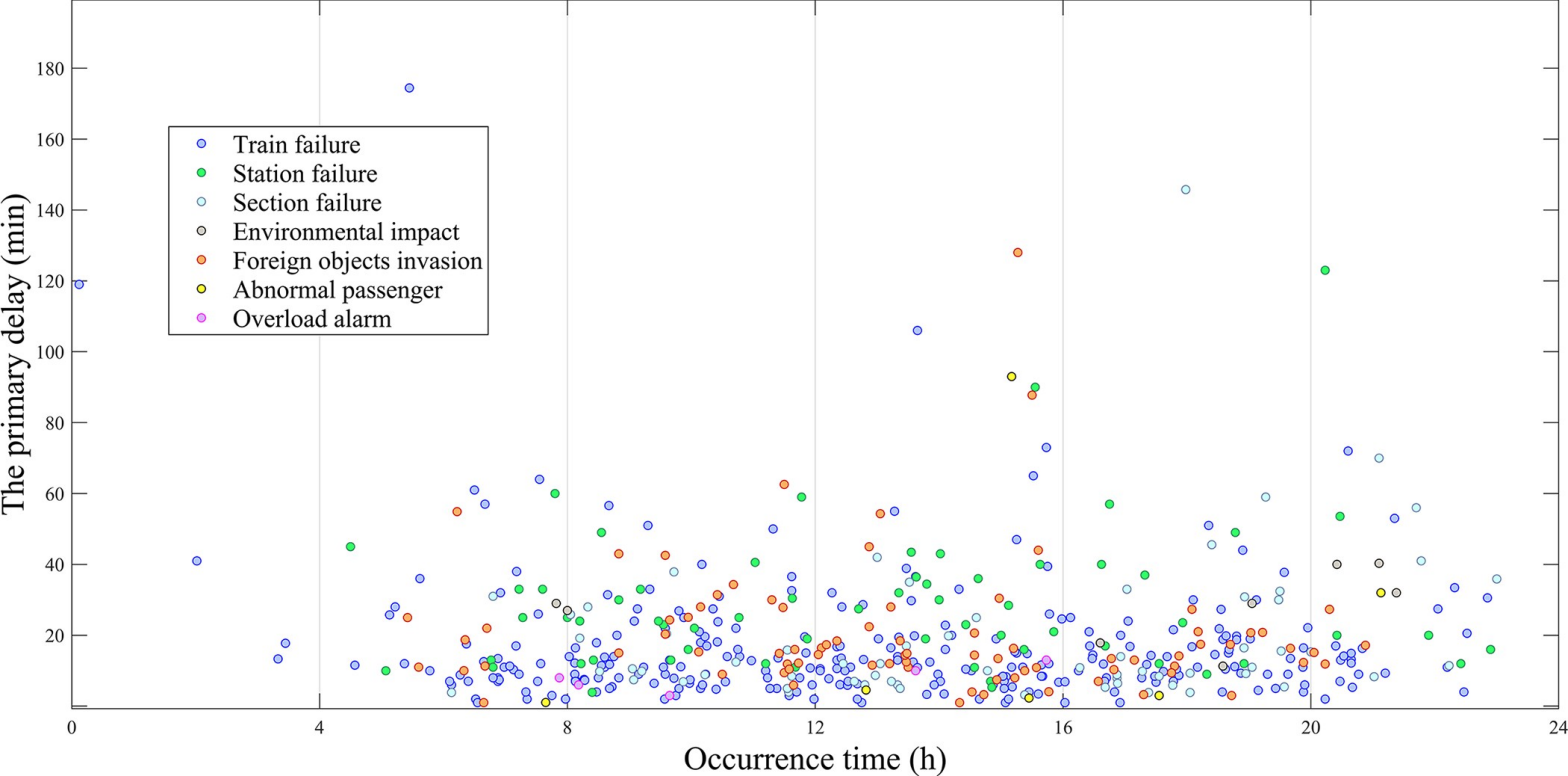
Distribution of primary delay samples based on occurrence time and event cause.

The distribution of the occurrence moments and causes of abnormal events is shown in Fig 9. In terms of the distribution of occurrence moments, most abnormal events are concentrated between 8:00 and 20:00. The number of abnormal events before 8:00 and after 20:00 is relatively small. Additionally, the average primary delay between 12:00 and 16:00 is higher compared to other periods. In the distribution of event causes, train failure has the highest frequency of occurrence, followed by the causes of foreign object intrusion, station and section equipment failures, which have the second highest frequency of occurrence. Other event causes have a lower frequency of occurrence.

The primary delays and the number of influenced trains under different event causes are analyzed in detail, as shown in Table 6. Among the seven types of abnormal event causes, the frequency of passenger abnormalities and overcrowding alarms is the smallest, at 1.2% and 1% respectively. This corresponds to the lowest average primary delay and number of influenced trains. The frequency of abnormalities caused by environmental influences is also very low, at about 1.6%, but it corresponds to a higher average primary delay and the highest average influence on the number of influenced trains. On-board train equipment failures have the highest frequency, at about 54.5%, corresponding to a lower average primary delay and influence. Station and section equipment failures and foreign objects invasion occur relatively less frequently. The average primary delay of station equipment failures as well as foreign object intrusion is similar, besides, the average number of trains influenced by section equipment failures and foreign object intrusion is also similar.

**Table 6:** Statistics of average primary delay and influenced train number under different event causes.

Event causes	Event number	Frequency	Average primary delay (min)	Average influenced train number
On-board equipment failure	273	54.49%	17.12	2.79
Station equipment failure	63	12.57%	28.59	5.71
Section equipment failure	65	12.97%	19.36	4.88
Environmental influence	8	1.60%	28.31	12.38
Foreign objects invasion	81	16.17%	20.59	5.02
Abnormal passenger	6	1.20%	22.65	1.17
Overload alarm	5	1.00%	8.00	1.20

## 4. Influence analysis of HSR delays

### 4.1. Analysis of influenced train number

From the above, it can be seen that emergent measures for abnormal events in the section mainly include temporary stopping and the combination of temporary stopping and speed limiting. Emergent measures for abnormal events in the station mainly include overtime stopping and delayed departure. Therefore, this section majorly explores the relationship between primary delays and influenced train number under different disposal measures for various types of section and station abnormal events.

By comparing and analyzing the applicability and advantages and disadvantages of four commonly used clustering algorithms, namely K-means, Fuzzy C-Means (FCM), Density Peaks Clustering (DPC), and Subtractive Clustering Method (SCM), this paper selects the Fuzzy C-Means that is more suitable for practical classification needs for clustering analysis. The primary delay of abnormal events and the service frequency of their spatiotemporal location, are used as the two basic indicators for clustering delay scenarios. Finally, in the sample dataset, 303 abnormal events in high-speed railway sections are clustered into 4 types, and 198 abnormal events at stations are clustered into 3 types. The clustering results and corresponding actual scenario parameter characteristics of abnormal events in sections and stations are shown in Table 7.

**Table 7:** Clustering results of abnormal events in high-speed railways.

Location	Type	Cluster center		Sample size	Ratio	Actual scenario parameter characteristics
		Normalization	Reality			
**Section**	1	(0.87, 0.65)	(7.68, 27.53)	87	28.7%	• Service frequency: 5~10 trains/h • Primary delay: 16~51 min
	2	(0.67, 0.48)	(4.31, 10.71)	78	25.7%	• The service frequency is concentrated in 2–6 trains per hour • The primary delay is concentrated between 3–20 minutes
	3	(0.26, 0.54)	(0.91, 15.33)	25	8.3%	• Service frequency: 0.1~2.5 trains/h • Primary delay: 1~40 min
	4	(0.89, 0.46)	(8.20, 9.70)	113	37.3%	• Service frequency: 6~11 trains /h • Primary delay: 2~17 min
**Station**	1	(0.81, 0.70)	(6.61, 29.23)	76	38.4%	• Service frequency: 4~10 trains /h • Primary delay: 14~65 min
	2	(0.38, 0.57)	(1.59, 15.16)	39	19.7%	• Service frequency: 0.1~3 trains /h • Primary delay: 1~45 min
	3	(0.80, 0.42)	(6.32, 6.79)	83	41.9%	• Service frequency: 3~11 trains /h • Primary delay: 1~14 min

For abnormal events in the sections, the proportion of type 4, type 1, and type 2 is relatively high, with corresponding proportions of 37.3%, 28.7%, and 25.7%, respectively; For abnormal events at the stations, the proportion of type 3 and type 1 is relatively high, with corresponding proportions of 41.9% and 38.4%, respectively. In terms of the distribution of parameter characteristics in service frequency and primary delay, each type of abnormal event has a significantly different main distribution range from other types of events, and the parameter characteristic value ranges of different types of abnormal events are both complementary and intersecting to some extent.

#### (1) Abnormal events in the sections

Based on the FCM clustering results, the distribution relationship between the primary delay and the number of influenced trains under the two main measures of each type of abnormal events corresponding to temporary section stopping and the combination of temporary section stopping and speed limiting are shown in Table 8. Among them, the delay propagation rate is defined as the number of influenced trains by the primary delay per minute under the corresponding abnormal event category and emergent response measures, train/min. The larger the ratio is, the faster the propagation rate of delay between trains will be.

**Table 8:** Analysis of the influence of sectional primary delays under major measures.

Type	1		2		3	4	
Major measures	Temporary stopping	Temporary stopping + Speed limiting	Temporary stopping	Temporary stopping + Speed limiting	Temporary stopping	Temporary stopping	Temporary stopping + Speed limiting
**Sample quantity**	48	21	47	22	17	66	26
**Delay propagation rate** **(train/min)**	0.16	0.22	0.14	0.17	0.06	0.16	0.23

For the third type of abnormal events in the section that adopt temporary stopping measures, although the corresponding data sample points have a large range of primary delays and the number of influenced trains, about 90% of the events generate primary de-lays less than 40 minutes, and will only influence one train, indicating that this type of event basically does not cause delay propagation between trains. At the same time, the average delay propagation rate corresponding to this type of event is the smallest among all events in the section, which means that for the third type of abnormal events in the section that adopt temporary stopping measures, the subsequent delay propagation rate between trains is the lowest.

#### (2) Abnormal events at stations

Based on the FCM clustering results, the number of samples and the average delay propagation rate corresponding to the main measures for each type of station abnormal events are shown in Table 9, and the distribution relationship between the primary delay and the number of influenced trains under the main measures is analyzed. Among them, the abnormal events in types 2 and 3 at stations that take measures for original departure delay only affect 1 to 2 trains, so there is basically no subsequent delay propagation between trains; for the abnormal events in type 1 at the station and the abnormal events in type 3 at the station that take measures for overtime station stopping, it is easy to form delay propagation between trains. Among them, the average primary delay of type 1 abnormal events affects 0.04 trains per minute, while the average primary delay of type 3 abnormal events that take measures for overtime station stopping affects 0.17 trains per minute, indicating that the delay propagation rate of type 3 is significantly higher than that of type 1.

**Table 9:** Analysis of the influence of stational primary delays under major measures.

Type	1		2		3	
Major measures	Overtime stopping	Delayed original departure	Overtime stopping	Delayed original departure	Overtime stopping	Delayed original departure
**Sample quantity**	26	14	7	12	49	11
**Delay propagation rate (train/ min)**	0.04	0.03	0.05	0.07	0.17	0.18

### 4.2. Analysis of maximum cumulative train delay

According to the records of the safety monitoring system, it is not possible to know the arrival delay distribution of the train that generates the primary delay at each station ahead, and the operation adjustment strategies of the influenced trains other than the primary influenced train are not recorded. In order to analyze the cumulative train delays under different scenarios of abnormal events in high-speed railways, this section studies the maximum cumulative train delay from the theoretical level. The cumulative train delay is defined as the sum of arrival delays of a train at current and forward stations. When the primary delay is short, the train is able to utilize the redundancy of the operating diagram to gradually absorb the delay, and the corresponding increase in the delay between train stations tends to 0. When the primary delay is long, the redundancy of the operating diagram is difficult to be completely absorbed, and the corresponding train delay propagation distance is longer, and the inevitable delay still exists when the train arriving at the terminal station.

The maximum cumulative train delay is estimated based on the train path and operational redundancy for the train that generates the primary delay. First, the number of stations that the train passes through during the period from the primary delay to the maximum cumulative delay is determined, based on which taking the smaller value between the theoretical number of influenced stations and the number of actual remaining stations on the train path. The corresponding calculation is:

$$m=min\left\{INT(\frac{{delay}_{1}}{{buf}_{a}+{buf}_{b}}),m_{re}\right\}$$ (13)

where *delay*_*1*_ is the primary station delay, min; *buf*_*a*_ is the average interval running redundancy on the current train path, min; *buf*_*b*_ is the redundancy of average stopping time on the corresponding train path, min; *m*_*re*_ is the total number of stations remaining ahead of the train’s path in the current dispatching segment.

According to the basic law of decreasing station arrival delays, the corresponding formula for calculating the maximum cumulative train delay is constructed based on the arithmetic sequence:

$${CD}_{max}=m\cdot {delay}_{1}-0.5m(m-1)({buf}_{a}+{buf}_{b})$$ (14)

Since the calculation of the maximum cumulative train delay is only for the first train that generates the primary delay, without considering other measures of over-traffic organization and stopping scheme changes, its primary delay can only be mitigated by the redundancy of section operation and stopping time redundancy, and the above formula is the corresponding theoretical maximum value. For the subsequent trains with knock-on delays, since the operation adjustment strategy of the subsequent trains is unknown, and the utilization of section redundancy needs to consider the adaptability of the scenario, this paper does not discuss the maximum cumulative delays of the knock-on delayed trains for the time being. At the same time, for trains that cross the scheduling section, it is necessary to update the corresponding delay and transportation parameters for calculation on the basis of projecting the delay of the incoming train that in dispatch section ahead of it.

**Fig 10:**
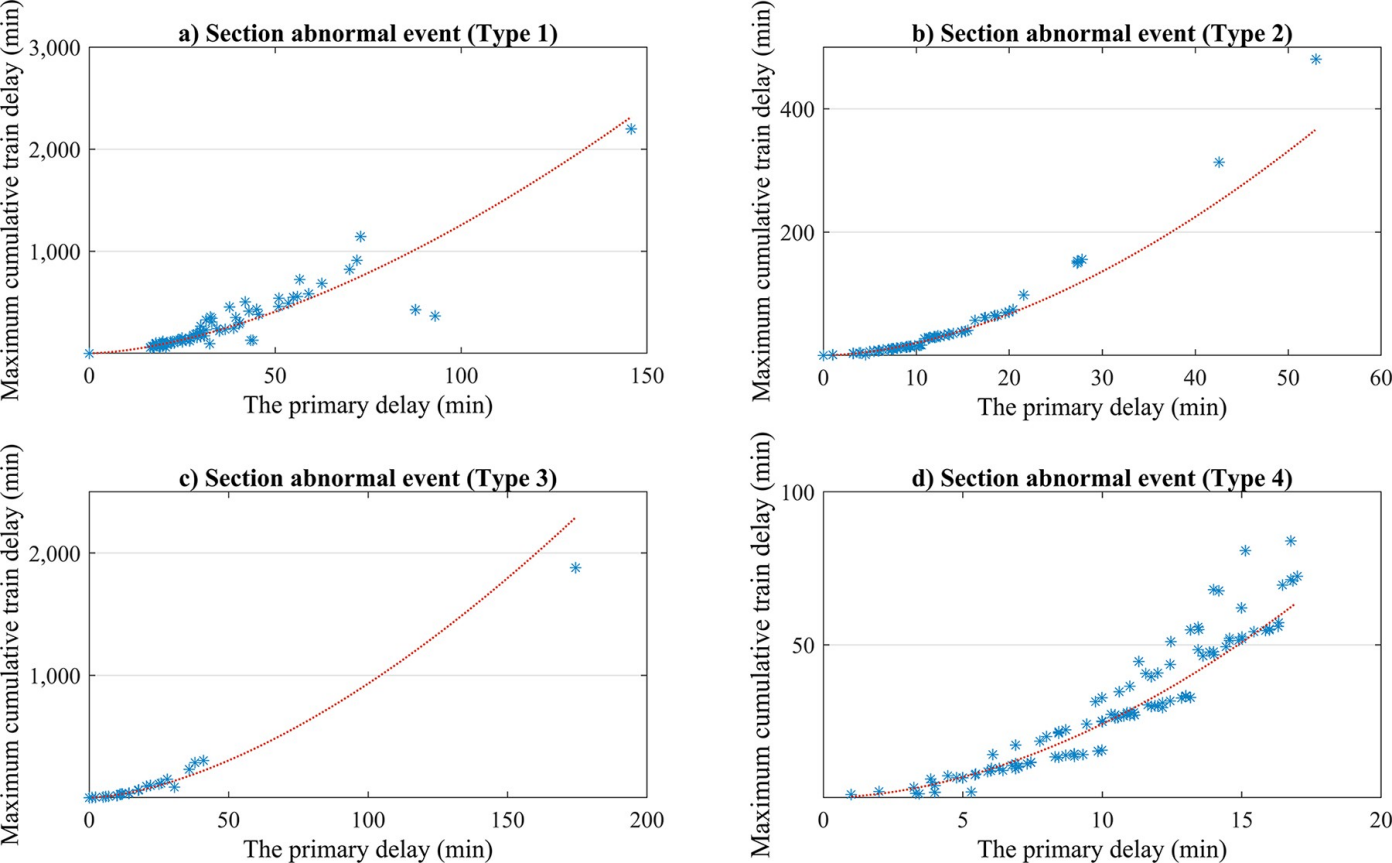
Distribution of maximum cumulative train delay for various abnormal events in the section.

**Fig 11:**
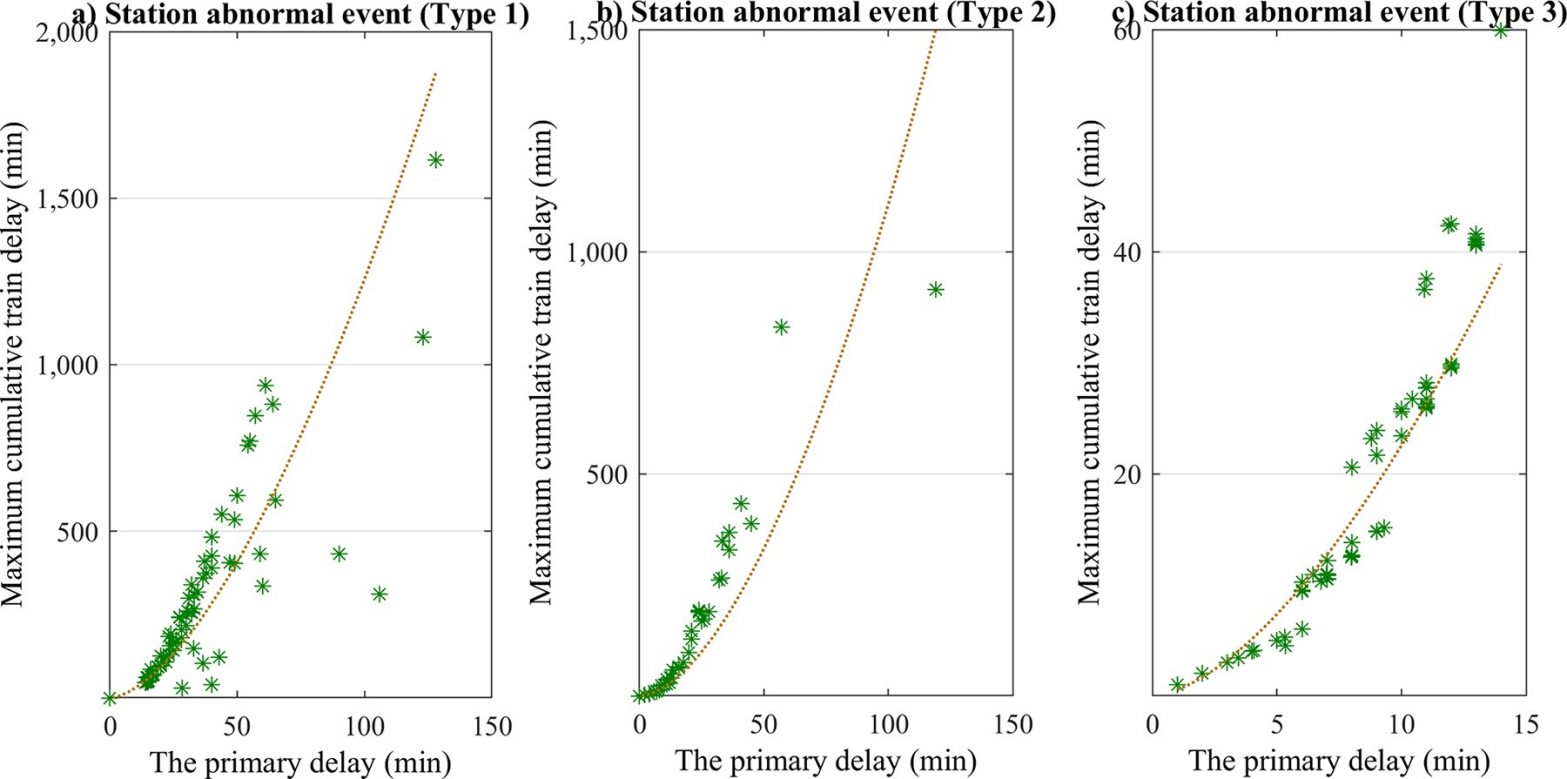
Distribution of maximum cumulative train delay for various abnormal events in the station.

The distribution of maximum cumulative train delay under various types of abnormal event scenarios in sections and stations are shown in Figs 10 and 11, respectively. By fitting the distribution relationship between the maximum cumulative train delay and the primary delay, the distribution relationship and determination coefficient *R*^*2*^ of the fitting curve under various abnormal event scenarios in the section and station are obtained, as shown in Table 10.

**Table 10:** The R^2^-distribution between the maximum cumulative train delay and primary delay for abnormal event scenarios.

Scenario	Abnormal event scenarios at the section				Abnormal event scenarios at the station		
Type	1	2	3	4	1	2	3
**Distribution**	Power law distribution	Power law distribution	Power law distribution	Power law distribution	Power law distribution	Power law distribution	Exponential distribution
**R** ^**2**^	0.8174	0.9956	0.9894	0.9115	0.6553	0.6756	0.9403

When the *R*^*2*^ is closer to 1, it indicates a higher correlation. According to the data in Table 10, it can be inferred that for trains experiencing primary delay, except for the third type of station abnormal event scenario that follows an exponential distribution, regardless of the type and location of the abnormal events, the relationship between the maximum cumulative train delay and primary delay obey a power-law distribution and have a high correlation at the theoretical level.

### 4.3. Analysis of the influence of scenario characterization parameters

In view of the technical requirement of minimum stopping time, the value of stopping time redundancy is less volatile, this paper mainly combines the effects of two scenario parameters, namely, section running redundancy and delay propagation rate, in order to study the distribution relationship between primary delay and cumulative train delay at stations, as well as the corresponding influence of delay propagation at the theoretical level.

**Fig 12:**
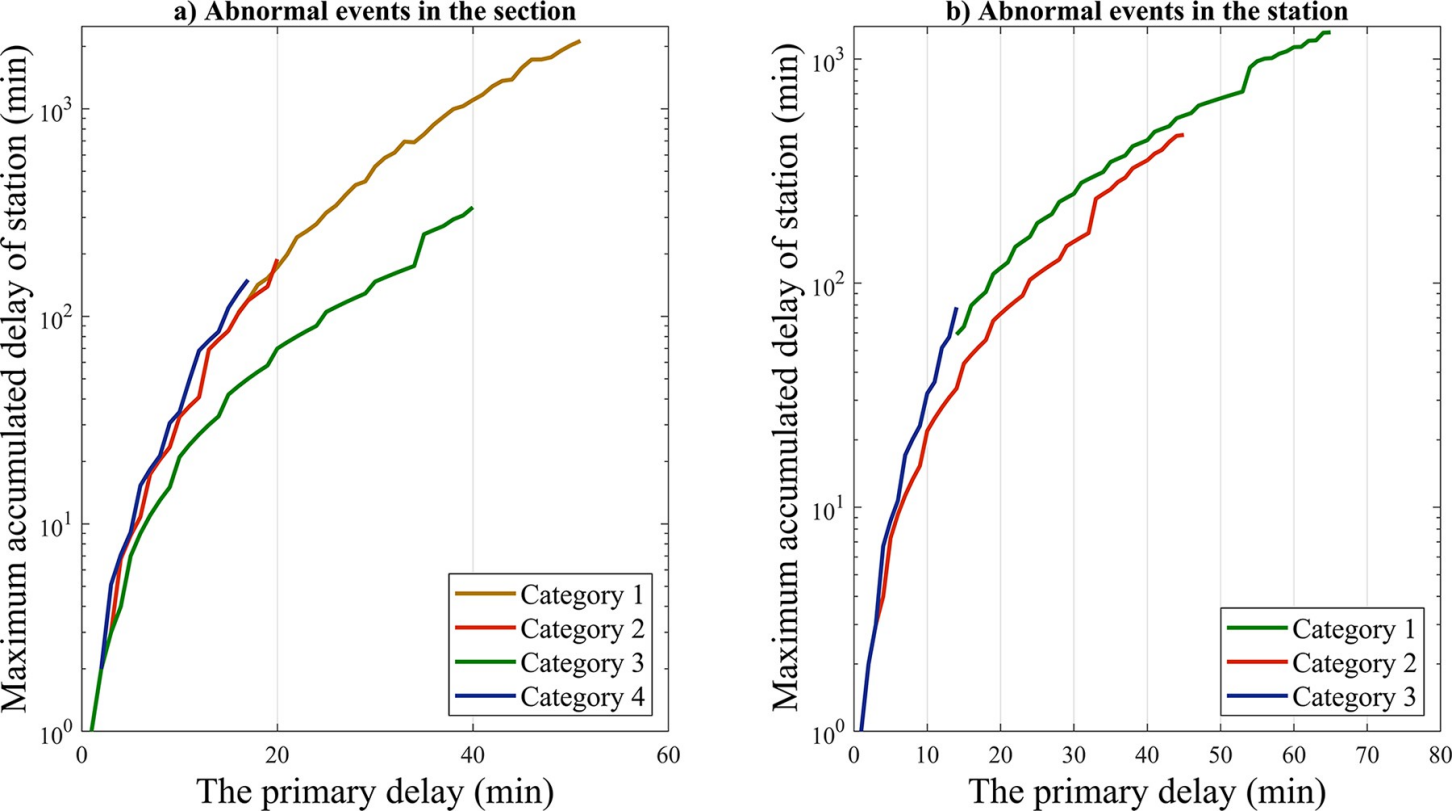
Accumulated station delay distribution under various abnormal event scenarios.

Based on the characteristics of the primary delay distribution, average section running redundancy and delay propagation rate corresponding to each type of abnormal events in sections and stations, the interaction relationship between the primary delay and the maximum cumulative train delay at stations in the corresponding scenarios is analyzed, and the specific distributions are shown in Fig 12. For abnormal events in the sections, the growth rates of cumulative delays for types 1, 2 and 4 tend to be consistent, where delays under type 1 events forms an extended complement to that of type 2; the growth rate of cumulative delays for type 3 is weaker than that of the other three types, which is due to its lower section operating redundancy and delay propagation rate. In addition, further analysis shows that the cumulative delay growth rate of abnormal events at stations is significantly lower than that abnormal event in sections as a whole, due to the lower delay propagation rate of abnormal events at stations.

**Fig 13:**
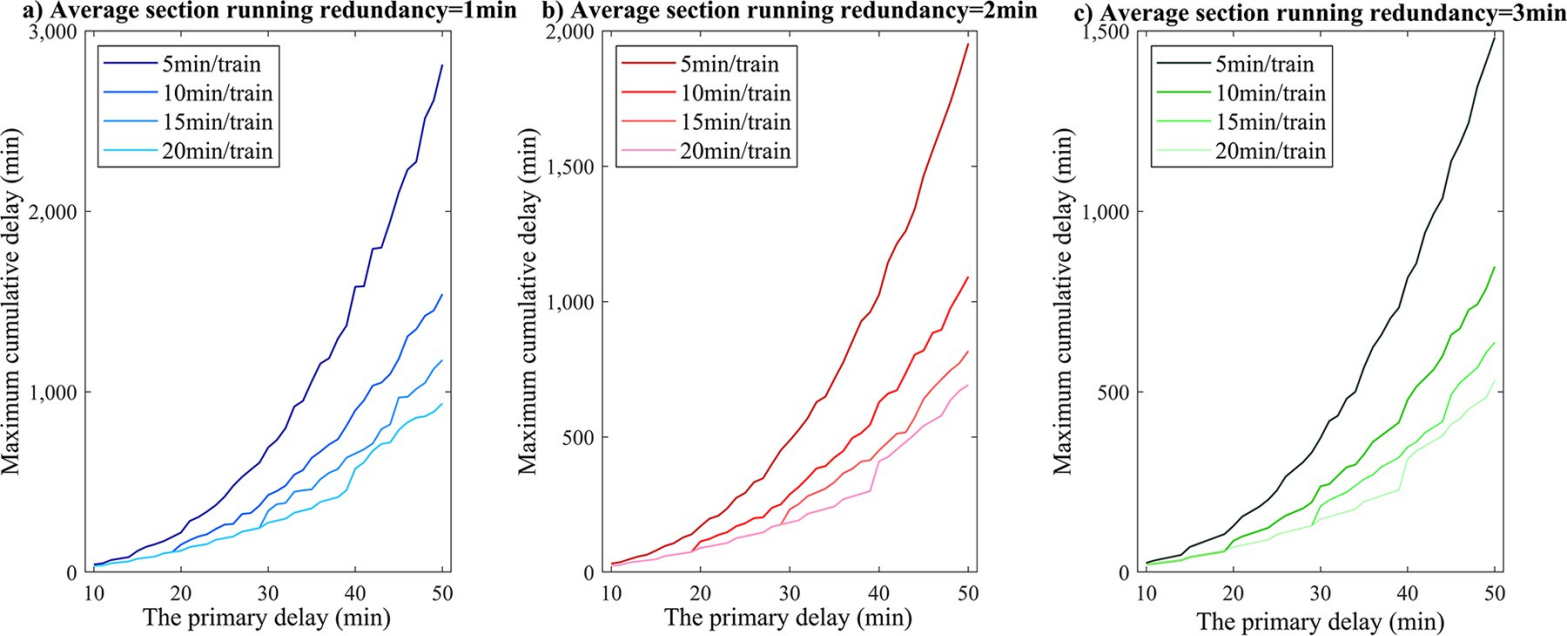
Changes in maximum cumulative station delay under different scenario parameter levels.

In order to further analyze the influence of the two characteristic parameters of section running redundancy and delayed propagation rate on the maximum cumulative train delay growth rate of stations, the primary delay ranging from 10 to 50 minutes is taken as the main target, as shown in Fig 13. The theoretical study results show that under the same primary delay condition, the higher the section running redundancy where the train is located is and the lower the train delay propagation speed will be, and the easier it is for the basic operation diagram to absorb part of the knock-on delays generated under the abnormal event.

### 4.4. Result discussion and strategy suggestion

#### 4.4.1. Result discussion

The main conclusions of the study are as follows:

(1) The proportion of primary delay time of less than 30 minutes reaches 90%. The number of abnormal event samples distributed in the sections is 1.53 times that of stations. Among them, for abnormal events in the section, temporary section stopping and combined temporary section stopping and speed limitation measures are mainly used, while for abnormal events at the station, the measures of overtime station stopping, delayed original departure and temporary section stopping are mainly used.
(2) In terms of occurrence time distribution, abnormal events are mostly concentrated in the period from 8:00 to 20:00, while the average primary delay during the period from 12:00 to 16:00 is relatively higher than other periods.
(3) As to the event cause distribution, the frequency of on-board train equipment failure is the highest, accounting for about 54.5%.
(4) Based on the FCM clustering results, abnormal events in the section are more likely to propagate than abnormal events at the station. For trains experiencing primary delay, regardless of the type and location of the abnormal events, the relationship between the maximum cumulative train delay and primary delay mostly obey a power-law distribution. Moreover, the values of R^2^ are all greater than 0.65, indicating a high correlation at the theoretical level.

## 4.4.2. Strategy suggestion

The delay of high-speed railway trains not only damages the interests of passengers, but also affects the operational efficiency and service level of the entire transportation system. The following suggestions are proposed:

(1) Reduce primary delay, comprehensively consider factors such as location and scenario parameter characteristics, and analyze the optimal emergency organization strategy for different scenarios.
(2) Scientifically and reasonably deploy operational redundancy, considering the busy time and space of route transportation, combined with the distribution of historical event delays, and optimizing reasonably on the basis of ensuring throughput capacity and efficiency.
(3) The number of arrival and departure tracks at the station has a significant impact on the delay propagation ratio parameter, and it needs to be set reasonably according to the actual situation.
(4) Considering the causes of the incident, the frequency of train failures is the highest, and it is necessary to strengthen regular maintenance and repair of equipment such as bogies and pantographs on trains. Timely and accurate identification and monitoring of foreign objects on the line should be carried out.

## 5. Conclusions

This paper studies the statistical distribution of primary delay, event location distribution, emergent measure distribution, occurrence time and event cause distribution, and other characteristics. Based on this research, the distribution characteristics of the influenced train number and the cumulative delay under kinds of disturbances have been discussed, where the effects of running redundancy, propagation rate, and related parameters on delay propagation have got quantitative analysis. In terms of delay data processing, this paper overcomes the interference of redundant data and conducts data filtering. In terms of parameter analysis, it considers the primary delay of correcting speed loss. The innovations and contributions of the paper are described from the following points:

(1) In terms of research objects, the paper comprehensively analyses the mechanism of primary delay and Knock-on delay, the propagation characteristics of delay in various emergency scenarios, and quantifies the impact of different scenario parameters such as operational redundancy and service frequency on delay propagation based on this.
(2) In terms of scenario feature parameter extraction, 9 scenario attributes including primary delay, service frequency and event causation were processed and extracted in detail. Considering the speed loss time under different emergency strategies, the primary delay data was corrected, and the service frequency of the transportation route was quantified in spatiotemporal segments.
(3) In terms of research methods, combining data statistics, cluster analysis, regression fitting, and numerical simulation can help to more accurately describe the characteristics of delay scenario parameters and the impact of delay propagation.
(4) At the application level, the relevant research results of this paper can provide reference for the applicability analysis of subsequent conflict resolution strategies, and provide a foundation for the reasonable assumptions, scenario parameter calibration, and constraint condition construction involved in the coordinated optimization model of train operation adjustment and delay propagation, thereby helping managers formulate more efficient high-speed railway operation organization and management plans.

## Supporting information

S1 File Raw data. (XLSX)

S2 File Multidimensional data of accidents. (XLSX)

S3 File Monthly accidents data. (XLSX)
